# Photocatalytic ATRP Depolymerization: Temporal Control at Low ppm of Catalyst Concentration

**DOI:** 10.1021/jacs.3c05632

**Published:** 2023-09-22

**Authors:** Kostas Parkatzidis, Nghia P. Truong, Krzysztof Matyjaszewski, Athina Anastasaki

**Affiliations:** † Laboratory of Polymeric Materials, Department of Materials, ETH Zurich, Vladimir-Prelog-Weg 5, Zurich 8093, Switzerland; ‡ Department of Chemistry, Carnegie Mellon University, 4400 Fifth Avenue, Pittsburgh, Pennsylvania 15213, United States

## Abstract

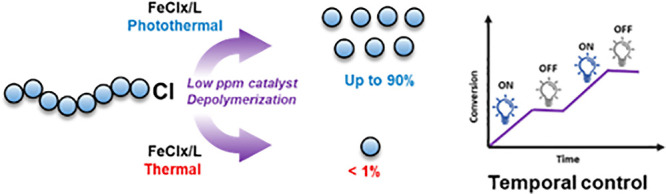

A photocatalytic ATRP depolymerization is introduced that significantly suppresses the reaction temperature from 170 to 100 °C while enabling temporal regulation. In the presence of low-toxicity iron-based catalysts and under visible light irradiation, near-quantitative monomer recovery could be achieved (up to 90%), albeit with minimal temporal control. By employing ppm concentrations of either FeCl_2_ or FeCl_3_, the depolymerization during the dark periods could be completely eliminated, thus enabling temporal control and the possibility to modulate the rate by simply turning the light “on” and “off”. Notably, our approach allowed preservation of the end-group fidelity throughout the reaction, could be carried out at high polymer loadings (up to 2M), and was compatible with various polymers and light sources. This methodology provides a facile, environmentally friendly, and temporally regulated route to chemically recycle ATRP-synthesized polymers, thus opening the door for further opportunities.

Reversible deactivation radical polymerization (RDRP) has enabled precise control over the molecular weight, molar mass distributions, sequence, architecture and end-group fidelity.^1,2^ In atom transfer radical polymerization (ATRP) and reversible addition–fragmentation chain-transfer (RAFT) polymerization, arguably the two most dominant controlled radical methodologies, this control is achieved by regulating the activation/deactivation equilibrium between active and dormant species.^3−5^ In recent years, significant efforts have been dedicated toward controlling the activation/deactivation equilibrium via external stimuli.^6−8^ Among them light has attracted significant attention, as it inherently possesses a number of unique properties and characteristics, such as high abundance, wide availability, and low cost, while it provides further possibilities for temporal and spatial control leading to its further implementation in 3D printing.^9−15^ In addition, photomediated polymerizations present significant advantages over traditional thermal approaches including faster reaction times, higher monomer conversions, and enhanced control over the molar mass distributions.^16−23^

Although the advantages of light have been carefully examined to efficiently catalyze controlled radical polymerizations, they have rarely been employed for the polar opposite: reversing controlled radical polymerization through depolymerization. The chemical recycling of polymers synthesized by RDRP methodologies has recently attracted considerable attention owing to the possibility to regenerate the starting monomer and subsequently use it to either reobtain the same polymer or an entirely new material.^24−26^ One notable advantage over more traditional pyrolysis approaches is the possibility to depolymerize at appreciably lower temperatures thanks to the active end-groups installed by RDRP. Aside from the undeniable sustainability benefits, intriguing mechanistic aspects can also be drawn. Currently, the vast majority of depolymerizations operate exclusively by using heat as an external stimulus.^27−32^ For example, Gramlich and co-workers explored the propensity of RAFT-synthesized macromonomer-based polymers to undergo depolymerization using trithiocarbonate as the RAFT agent.^33^ In 2022, our group expanded the scope of thermal RAFT depolymerization to include nonbulky polymers such as poly(methyl methacrylate) regenerating the monomer at a high yield under thermodynamically favorable conditions.^34,35^ A year later, Sumerlin’s group and our group independently demonstrated the possibility to accelerate depolymerizations in the presence of either visible or UV irradiation.^36,37^ However, in both instances the contribution of thermal depolymerization was very prominent (i.e., high depolymerization conversions could be achieved even in the absence of light), and as such temporal regulation was not possible. In the ATRP arena, Raus first showed that during the polymerization of macromonomers, significant depolymerization could be detected even at relatively low temperatures, thus prohibiting high polymerization conversions.^38^ Matyjaszewski’s group also demonstrated the depolymerization of bulky polymers at 170 °C using copper catalysis.^39^ Ouchi and co-workers were the first to enable the thermal depolymerization of PMMA by utilizing a ruthenium catalyst, recovering up to 24% of monomer.^40^ Matyjaszewski and co-workers also reported successful thermal depolymerization of nonbulky polymers using either copper or iron catalysis at 170 °C.^41,42^ Nevertheless, high monomer conversions could not be reached due to the significant loss of end-group observed at high depolymerization temperatures. Preliminary efforts to use light for depolymerization were recently conducted by Yagci’s group using dimanganese decacarbonyl, albeit leading to low conversions (<20%) and detrimental side reactions.^43^ As such, an efficient photocatalytic ATRP depolymerization enabling temporal control and high monomer conversions remains elusive.

In this work, we develop a photocatalytic ATRP depolymerization method using iron catalysis, and the highlights of our approach are presented in Figure 1.

**Figure 1:**
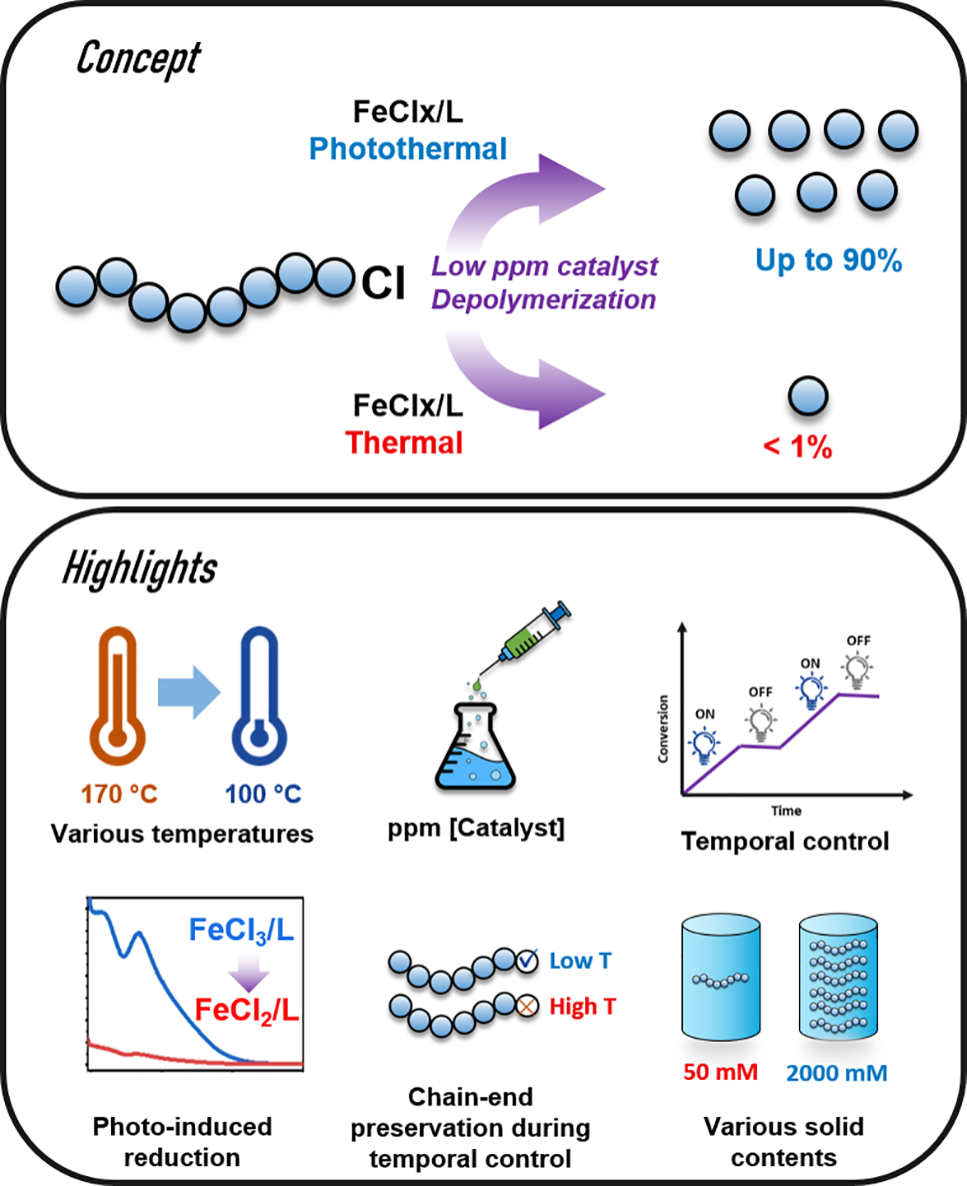
Schematic illustration and highlights of photocatalytic depolymerization.

Poly(benzyl methacrylate) (PBzMA) was synthesized via an optimized activator regenerated by electron transfer (ARGET) ATRP approach (*Đ* ≈ 1.15, Figures S2 and S3, Table S2) and was subsequently used for our depolymerization studies. In search of a suitable photocatalytic ATRP depolymerization system, our efforts were directed to iron catalysis using FeCl_2_, due to the low catalyst toxicity and cost.^44,45^ PBzMA was then subjected to judiciously optimized depolymerization conditions (Figures S5–S7, Tables S3–S7) employing stoichiometric amounts of catalyst (i.e., 1 equiv of FeCl_2_ with respect to the halogen end-group) under blue light irradiation at 170 °C. Blue light irradiation was selected as the ideal wavelength for iron-based catalysts as previously demonstrated by the polymerization literature.^46^ Within 5 min of reaction, almost 90% of monomer was successfully regenerated, as confirmed by ^1^H NMR spectroscopic analysis, without any significant change in the polymers’ molecular weight and *Đ* (Figure 2a and b, Table S4). To date, this is the highest depolymerization yield reported for ATRP-synthesized polymers. However, the control experiment in the absence of light irradiation (i.e., using only heat) revealed only slightly lower yield (i.e., 88%), thus suggesting that temporal control under these conditions would not be feasible due to noticeable contribution of thermal depolymerization. To address this, we gradually decreased the depolymerization temperature from 170 °C to 150 and 120 °C (Figure 2b). Although photothermal depolymerization again reproducibly gave slightly higher yield as opposed to the exclusively thermal system, the significant extent of depolymerization observed under heat still prohibited the possibility of temporal control. However, the fact that 120 °C still resulted in appreciable depolymerization yield (i.e., 71%) was very encouraging, as previous reports reached comparable yields at much higher temperatures (e.g., 170 °C).^41,42^ Notably, at 100 °C the thermal depolymerization was significantly suppressed with only 6% of yield achieved within comparable timeframes (Figure 2b). Intrigued by these data, we subsequently investigated the possibility of “on/off” temporal control during depolymerization using intermittent light and dark exposure. During the first period of light irradiation (i.e., 20 min), 18% of depolymerization yield was attained (Figure 2c, Table S8). However, upon switching the light “off”, the depolymerization continued at a comparable rate, reaching 31% of yield within another 20 min. Initially, we were perplexed by the lack of temporal control in this system, as the control thermal experiment revealed only minimal yield in the absence of light irradiation. The lack of temporal control was attributed to the high concentration of polymer radicals generated by the FeCl_2_ activator which, upon switching the light “off”, may act as reducing agents of the in-situ-formed FeCl_3_ thereby resulting in the continuation of the depolymerization.

**Figure 2:**
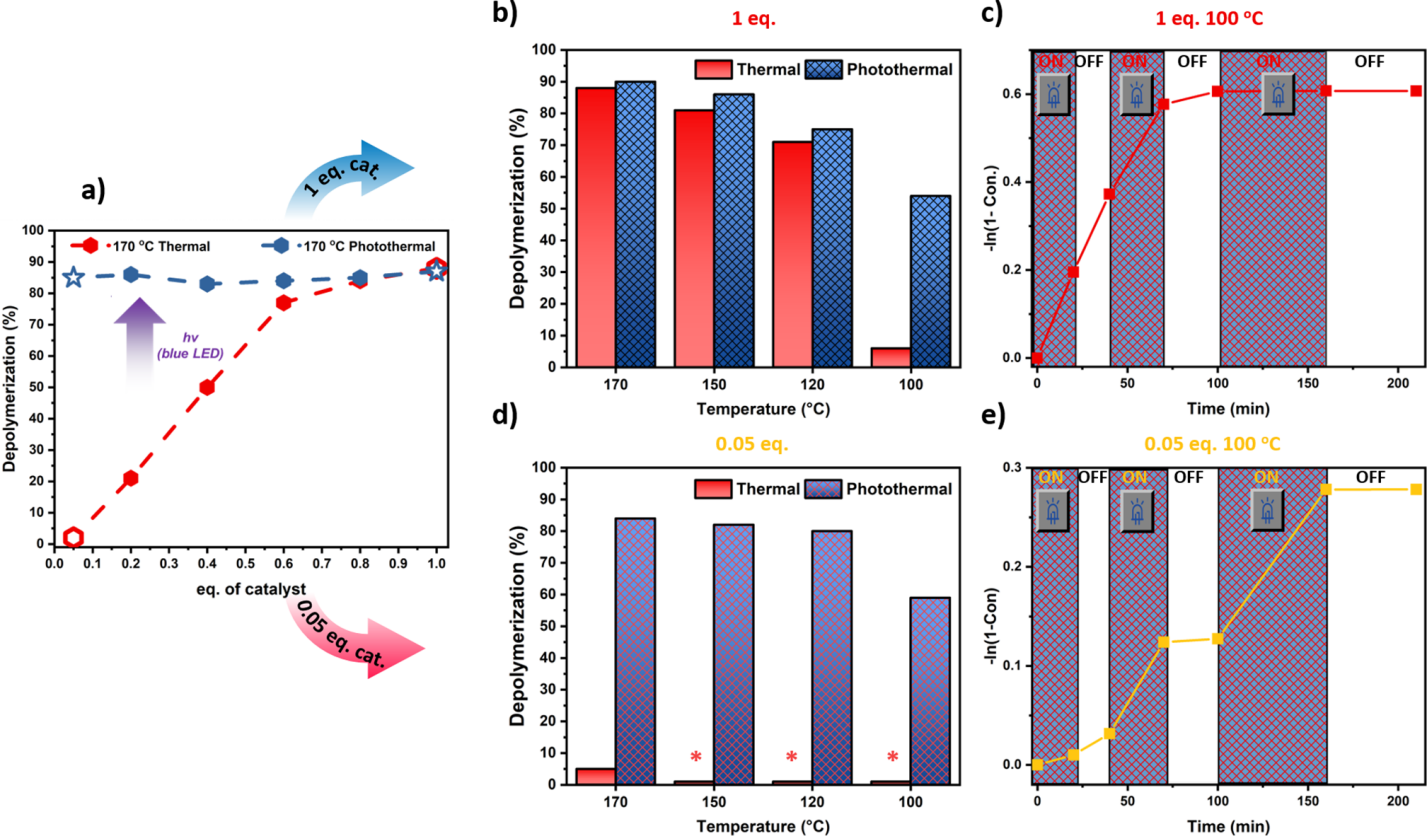
(a) Thermal (red) and photothermal (blue) depolymerization of PBzMA at different catalyst concentrations. (b and d) Comparison of thermal and photothermal depolymerization of PBzMA at different temperatures using 1 and 0.05 equiv of catalyst. (c and e) Temporal control of depolymerization of PBzMA at 100 °C using 1 and 0.05 equiv of catalyst.

Inspired by previous works in photomediated RDRPs, we envisioned that lowering the catalyst concentration may lead to enhanced temporal control.^47−49^ Our hypothesis was that by significantly reducing the catalyst concentration, we will not only lower the amount of active chains at a given time but also completely eliminate the thermal depolymerization. Indeed, when 0.05 equiv of FeCl_2_ was employed, a pronounced contribution of light was already evident even at 170 °C whereby only 5% of yield was observed in the absence of irradiation (Figure 2a and d). Instead, photothermal depolymerization at 170 °C yielded 84% of BzMA. To the best of our knowledge, this is the highest depolymerization yield achieved in the presence of ppm catalyst concentration, as previous strategies reported lower yields while employing up to 200 times higher catalyst loadings.^42^ The high depolymerization yields achieved are attributed to the use of light as an external stimulus, with the proposed mechanism depicted in Scheme 1. FeCl_2_ activates the chain-end-forming FeCl_3_ and enables the unzipping of the polymer chain. Blue light irradiation then enables the continuous reduction of FeCl_3_ back to FeCl_2_, thus facilitating an efficient depolymerization equilibrium.^46,50^

**Scheme 1:**
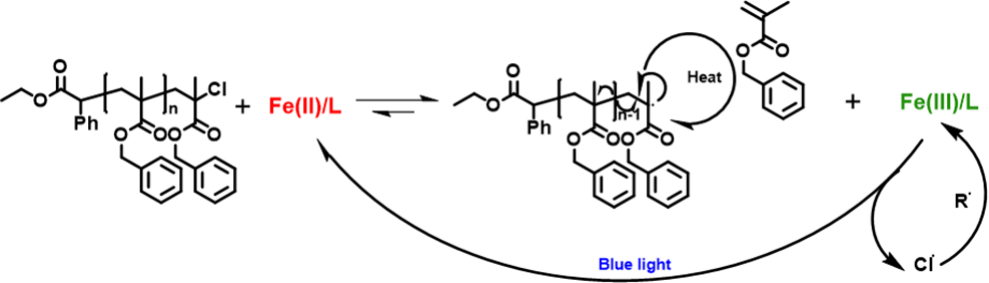
Proposed Mechanism of Photocatalytic ATRP Depolymerization

Notably, by further lowering the temperature to 150, 120, and 100 °C, the thermal depolymerization could be completely eliminated, thus indicating that temporal control should now be feasible. Indeed, an improved temporal control was observed, as shown in Figure 2e (Table S9). For instance, by switching the reaction “off” at 70 min, a complete discontinuation of the depolymerization was observed. On re-exposing the mixture to light irradiation, the original depolymerization rate was restored. The slightly imperfect temporal control observed at the very early depolymerization stages (<5% of total yield) was attributed to a small amount of radicals generated by the activator. Collectively, our data show that by utilizing ppm concentrations of FeCl_2_ at low temperatures (i.e., 100 °C), enhanced temporal control can be attained. Figure 2a further highlights the superiority of photocatalytic depolymerization, as very high conversions can be achieved regardless of the catalyst concentration employed. Instead, thermal depolymerizations can only achieve high yields at much higher catalyst loadings (at least 16 times higher).

To develop a more user-friendly photothermal depolymerization protocol, we were subsequently interested in replacing the FeCl_2_ activator with an FeCl_3_ deactivator. FeCl_3_ is a more air-stable precatalyst, thus further simplifying our approach. In addition, starting the reaction directly with the deactivator may further suppress depolymerization during the “dark” periods, as the amount of active FeCl_2_ will be even more limited. Kinetic experiments of FeCl_2_ versus FeCl_3_ were first conducted under identical concentrations with FeCl_3_ exhibiting a slightly lower depolymerization rate following an initial induction period (Figure 3a, Table S10), as expected from the polymerization literature. Photothermal depolymerization of PBzMA at 170 °C led to approximately 70% of depolymerization with the control experiment revealing only 5% of yield in the absence of irradiation (Figure 3b, Figures S8 and S9, Tables S11 and S12).

**Figure 3:**
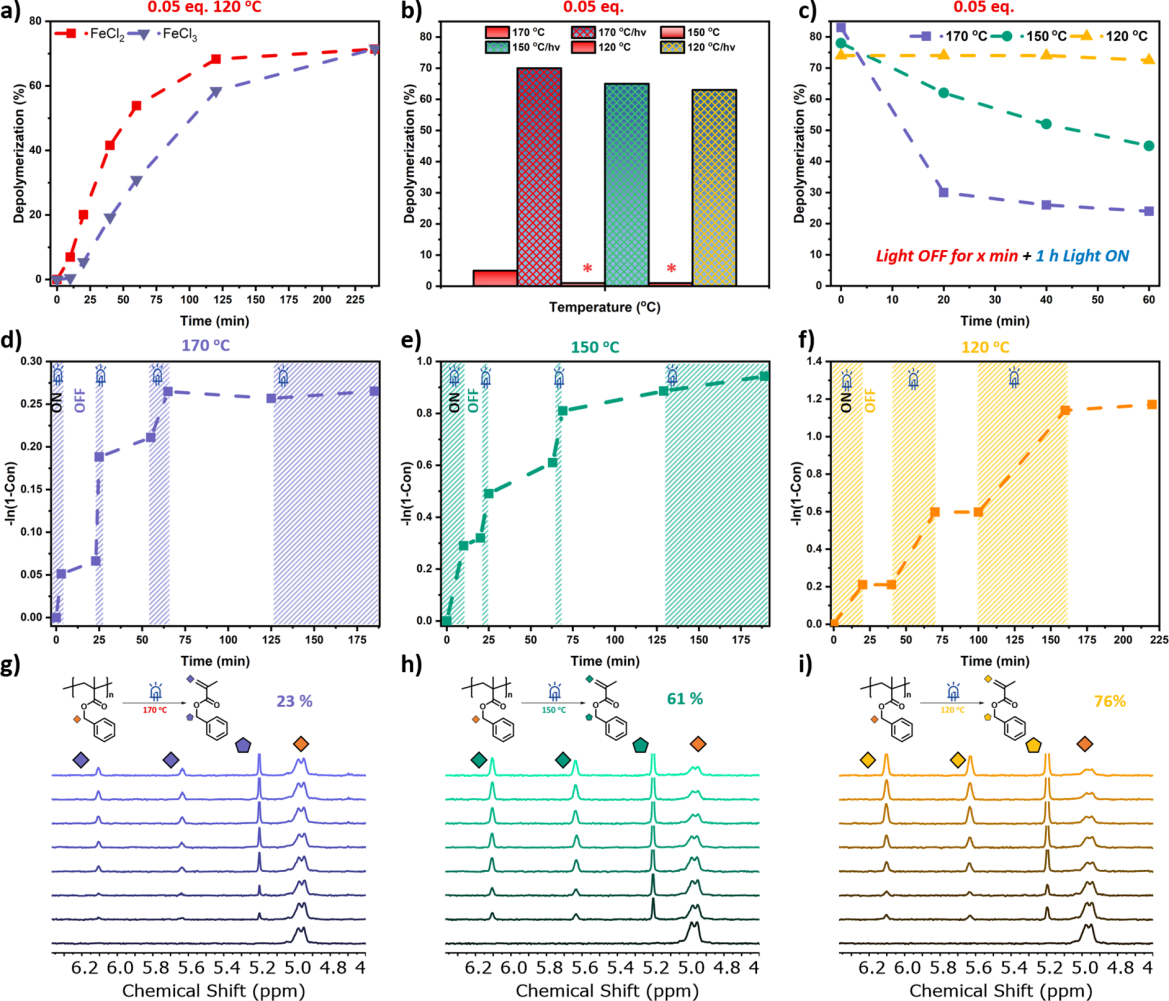
(a) Comparison of depolymerization kinetics utilizing FeCl_2_ (red) and FeCl_3_ (purple) catalyst. (b) Comparison of thermal and photothermal depolymerization of PBzMA at different temperatures using 0.05 equiv of catalyst (FeCl_3_). (c) Incubation experiments at different temperatures. (d–f) Temporal control of depolymerization of PBzMA at 170, 150, and 120 °C using 0.05 equiv of catalyst. (g–i) ^1^H NMR spectra of the temporal control experiments.

Instead, no monomer regeneration was observed under exclusively thermal depolymerization at either 150 or 120 °C, perhaps suggesting that better temporal control can be achieved under these temperatures. Although “on/off” experiments via photothermal depolymerization at 170 and 150 °C were moderately successful at yielding higher “on” periods than the ones at 120 °C, the recommended temperature for ideal temporal control is 120 °C. Figure 3c shows that a photothermal depolymerization at 170, 150, or 120 °C gives approximately similar yields in the absence of “off” cycles. However, for each “off” cycle at either 150 or 170 °C, the final depolymerization yield is significantly lowered. For example, at 170 °C by keeping the light “off” for 20 min, followed by a prolonged light “on” period, only 30% of yield can be reached. Instead, by continuously irradiating the reaction mixture (i.e., without the initial “off” period), much higher yields were attained (>80%). These results suggest that increasing the depolymerization temperature led to a significant loss of end-group (Figure 3d−i, Tables S13–S17), in line with previous reports.^39,41,42^

In contrast, photothermal depolymerizations at 120 °C are completely unaffected by the “off” periods and equally high overall yields can be obtained despite several light/dark cycles. Figure 3f shows our optimal data whereby excellent temporal control can be observed throughout the depolymerization without compromising the final yield. The reaction ceases during the “off” cycles regardless of the duration of these periods (20, 30, 60 or even 120 min), which further highlights the good temporal control obtained in this system (Figure S10). To expand the scope of our approach, we also showed that it is compatible with different polymers such as poly(*n*-butyl methacrylate) and poly(methyl methacrylate) with similar yield as in the case of PBzMA (Table S18). Additionally, we also employed green light for the depolymerization, which resulted in slightly lower depolymerization yields (Table S19). This is in line with the polymer literature, whereby green light also resulted in lower polymerization conversions, with the absorption spectra of the iron-based catalysts showing greater overlap in the emission spectra of blue LED when compared to green LED.^46^ For a more detailed optimization and to identify the optimal wavelength, one should conduct action plots in order to thoroughly investigate the effect of each specific wavelength on the depolymerization performance and employ the most suitable wavelength for each selected iron-based catalyst.^51^ Last but not least, we examined the possibility of the developed photothermal depolymerization to operate under higher concentrations (so far the experiments were performed at 50 mM repeating unit, which corresponds to 9 mg/mL of polymer concentration) (Table S20). Notably, even at 2 M concentration of repeating unit, 50% of yield could be obtained, thus highlighting the robustness of the system. It is noted that this ATRP photocatalytic depolymerization can operate at higher concentrations when compared to previous photo-RAFT depolymerization approaches.^36,37^

To summarize, we have demonstrated an efficient photocatalytic ATRP depolymerization using very low concentrations of low-toxicity FeCl_2_ or FeCl_3_. By lowering the reaction temperature from 170 °C to 120 or 100 °C, thermal depolymerization was successfully eliminated, thus allowing regulation of the depolymerization via intermittent “on/off” cycles. Importantly, under our judiciously optimized conditions, we were able to preserve the end-group fidelity throughout the reaction, enabling temporal regulation regardless of the amount of “on/off” cycles conducted. This depolymerization methodology offers a facile chemical recycling approach to reach near quantitative monomer yields while also enabling temporal regulation. We believe that our work can open new avenues, for example in the field of reversed additive manufacturing and lithography.^31^

## Supporting Information Available

General information, experimental procedures, H NMR spectra, SEC traces. (PDF)
