# Supplementary material for: Photocatalytic ATRP Depolymerization: Temporal Control at Low ppm of Catalyst Concentration
Source: J Am Chem Soc. 2023 Sep 22;145(39):21146–51. doi: 10.1021/jacs.3c05632 (PMC10557129; doi:10.1021/jacs.3c05632)
Supplement: Supplementary file 1 — ja3c05632_si_001.pdf [file ja3c05632_si_001.pdf]

Supporting information

Photocatalytic ATRP Depolymerization: Temporal Control at Low ppm of Catalyst Concentration

Kostas Parkatzidis, Nghia P. Truong, Krzysztof Matyjaszewski*, Athina Anastasaki*

Laboratory of Polymeric Materials, Department of Materials, ETH Zurich , Vladimir–Prelog–Weg 5, Zurich 8093, Switzerland

Department of Chemistry, Carnegie Mellon University , 4400 Fifth Avenue, Pittsburgh, Pennsylvania, 15213, United States.

Materials

All materials were purchased from Sigma Aldrich or Fischer Scientific and used as received unless otherwise stated. All monomers were purified by passing through a column of basic alumina before being used.

^{1}H NMR spectra were recorded on a Bruker DPX-300 spectrometer in deuterated chloroform ($CDCl_3$). Chemical shifts are given in ppm downfield from tetramethylsilane referenced to residual $CHCl_3$ protons. Monomer conversions were determined via proton ^{1}H-NMR spectroscopy by comparing the integrals of monomeric vinyl protons to monomer and polymer signals.

Size exclusion chromatography (SEC) analysis of polymer samples was performed using a Shimadzu modular system comprising a CBM-20A system controller, an SIL-20A automatic injector, a 10.0 μm beadsize guard column (50 × 7.5 mm) followed by three KF-805L columns (300 × 8 mm, bead size: 10 μm, pore size maximum: 5000 Å), an SPD-20A ultraviolet detector, and an RID-20A differential refractive-index detector. The temperature of the columns was maintained at 40 °C using a CTO-20A oven. The eluent was N,N-dimethylacetamide (HPLC grade, with 0.03% w/v LiBr) and the flow rate was kept at 1 mL min^{-1} using an LC-20AD pump. A molecular weight calibration curve was produced using commercial narrow molecular weight distribution poly(methyl methacrylate) standards with molecular weights ranging from 5000 to 1.5×10^6. Samples were filtered through 0.45 μm filters prior to injection.

Photoreactor Characteristics

The homemade photoreactor was built into a tube-like shape (diameter of 12 cm and height of 15 cm). On the inner walls of the photoreactor, an LED strip (Mxellex®) was placed inside in a spiral pattern. The final number of LEDs inside the photoreactor was 70. A silicone oil bath (diameter of 5.5 cm and height of 5 cm) where the reaction was heated was placed in the center of the photoreactor (**Figure S3**). Aluminum foil was used to cover the top of the reactor from external light. Photoreactor emission spectra were collected on a Varian Cary Eclipse spectrometer (**Figure S1**). The maximum emission wavelengths for blue and green are 460 nm (±17) and 510 nm (±31), respectively. Photoreactor intensity was determined using a Thorlabs PM100D light power meter, equipped with a S170C Microscope Slide Power Sensor (**Table S1**).

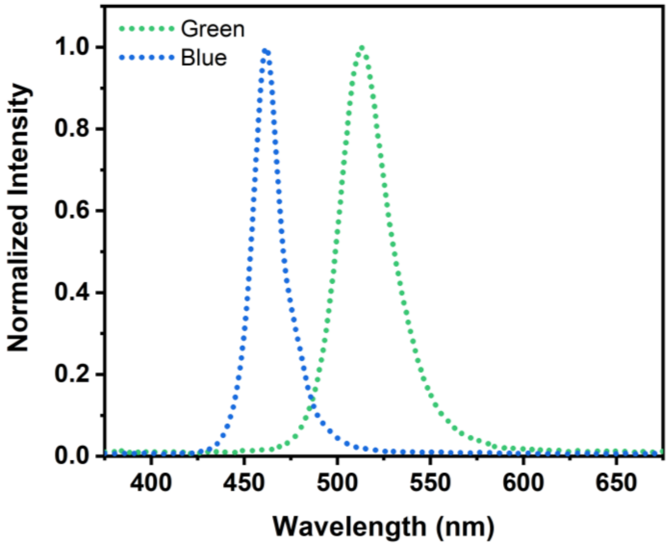

Figure S1: Normalized emission intensity of the blue and green LED strip used for the photoreactor.

Table S1:Characterization data of the photoreactor.

**Reactor**	**Wavelength (nm)**	**FWHM (nm)**	**Intensity (mW/cm^2)**
Blue	460	17	7.26 ± 0.55
Green	510	31	4.62 ± 0.14

General procedure for polymer synthesis

ARGET ATRP was used for the synthesis of the polymers (**Scheme S1**).

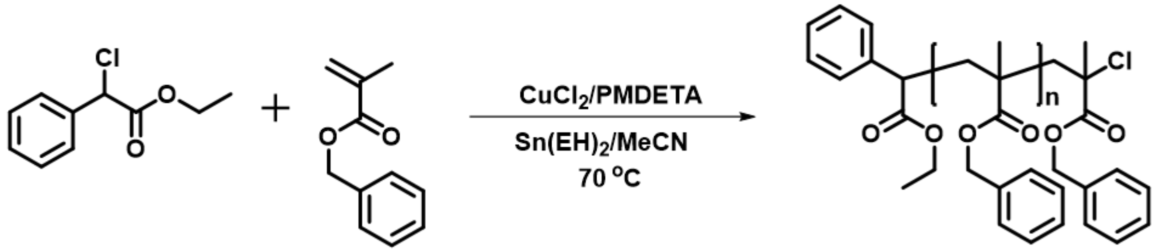

Scheme S1: Chemical illustration of polymerization of benzyl methacrylate via ARGET ATRP under the following conditions: [ECPA]:[BzMA]:[$CuCl_2$]:[PMDETA]:[$Sn(EH)_2$] = [1]:[100]:[0.2]:[0.2]:[0.08] in MeCN (1:1.25 solvent to monomer volume ratio) at 70 °C.

In a round bottom flask, equipped with a stirring bar, 59.5 mg of $CuCl_2$ (0.44 mmol, 0.15 equiv.) was dissolved in acetonitrile (40 mL) alongside 123.2 µl of N,N,N′,N′′,N′′-pentamethyldiethylenetriamine (PMDETA) (0.59 mmol, 0.2 equiv.). The solution was sonicated for 1 min to ensure full solubilization of the copper salt, resulting in a light blue solution. Next, 506.7 µl of ethyl α-chlorophenylacetate (ECPA) (2.95 mmol, 1 equiv.) and 50 ml benzyl methacrylate (BzMA) (0.295 mol, 100 equiv.) were added to the flask, which was sealed with a rubber septum and deoxygenated via nitrogen purging for 20 min. After deoxygenation, 76.45 µl of pre-deoxygenated tin(II) 2-ethylhexanoate ($Sn(EH)_2$) (0.24 mmol, 0.08 equiv.) was added, and the flask and the color of the solution immediately intensified resulting in indigo blue color solution. Finally, the reaction flask was immersed in pre-heated oil bath at 70 °C allowed to polymerize under constant stirring. The reaction was stopped after 2h by opening it to air. Samples were taken to measure ^1H NMR and SEC, and the rest of the polymerization solution was diluted with acetone and passed through a basic alumina column to remove the catalyst. The solution was concentrated via solvent evaporation and precipitated into cold methanol. The isolated polymer was dried in a vacuum oven at room temperature over the weekend. The purified polymer was analyzed again with ^1H NMR and SEC to ensure its purity and final molecular weight and dispersity.

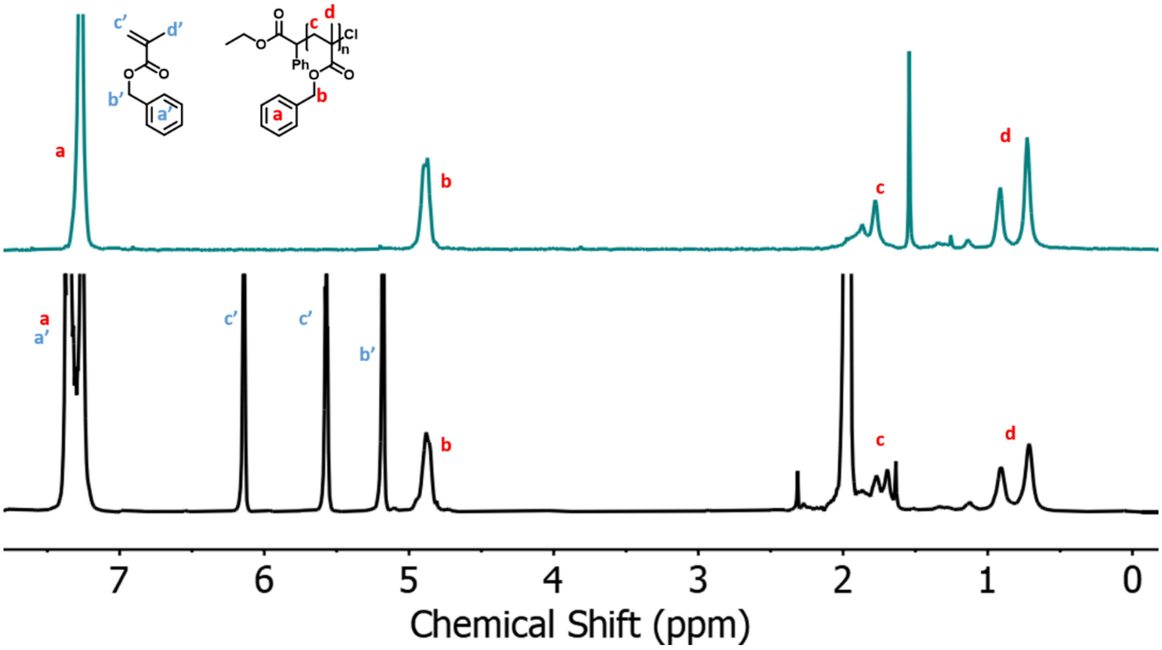

Figure S2: ^{1}H NMR spectra of poly(benzyl methacrylate) before (bottom) and after (top) purification, synthesized via ARGET ATRP using the following conditions: [ECPA]:[BzMA]:[$CuCl_2$]:[PMDETA]:[$Sn(EH)_2$] = [1]:[100]:[0.2]:[0.2]:[0.08] in MeCN (1:1.25 solvent to monomer volume ratio) at 70 °C.

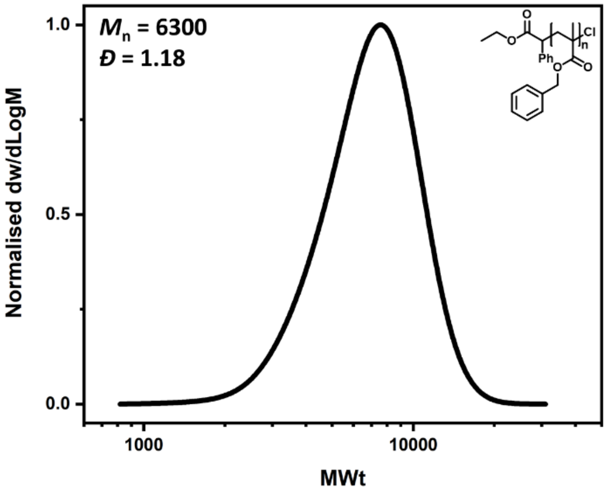

Figure S3: SEC trace of purified poly(benzyl methacrylate) synthesized via ARGET ATRP using the following conditions: [ECPA]:[BzMA]:[$CuCl_2$]:[PMDETA]:[$Sn(EH)_2$] = [1]:[100]:[0.2]:[0.2]:[0.08] in MeCN (1:1.25 solvent to monomer volume ratio) at 70 °C.

Table S2: Polymers investigated in this study. All the monomers have been polymerized following the general polymerization procedure.

Polymer	M_n	$Đ$
$PBzMA_{39}$	6300	1.15
$PnBMA_{38}$	5000	1.28
$PMMA_{51}$	5500	1.15

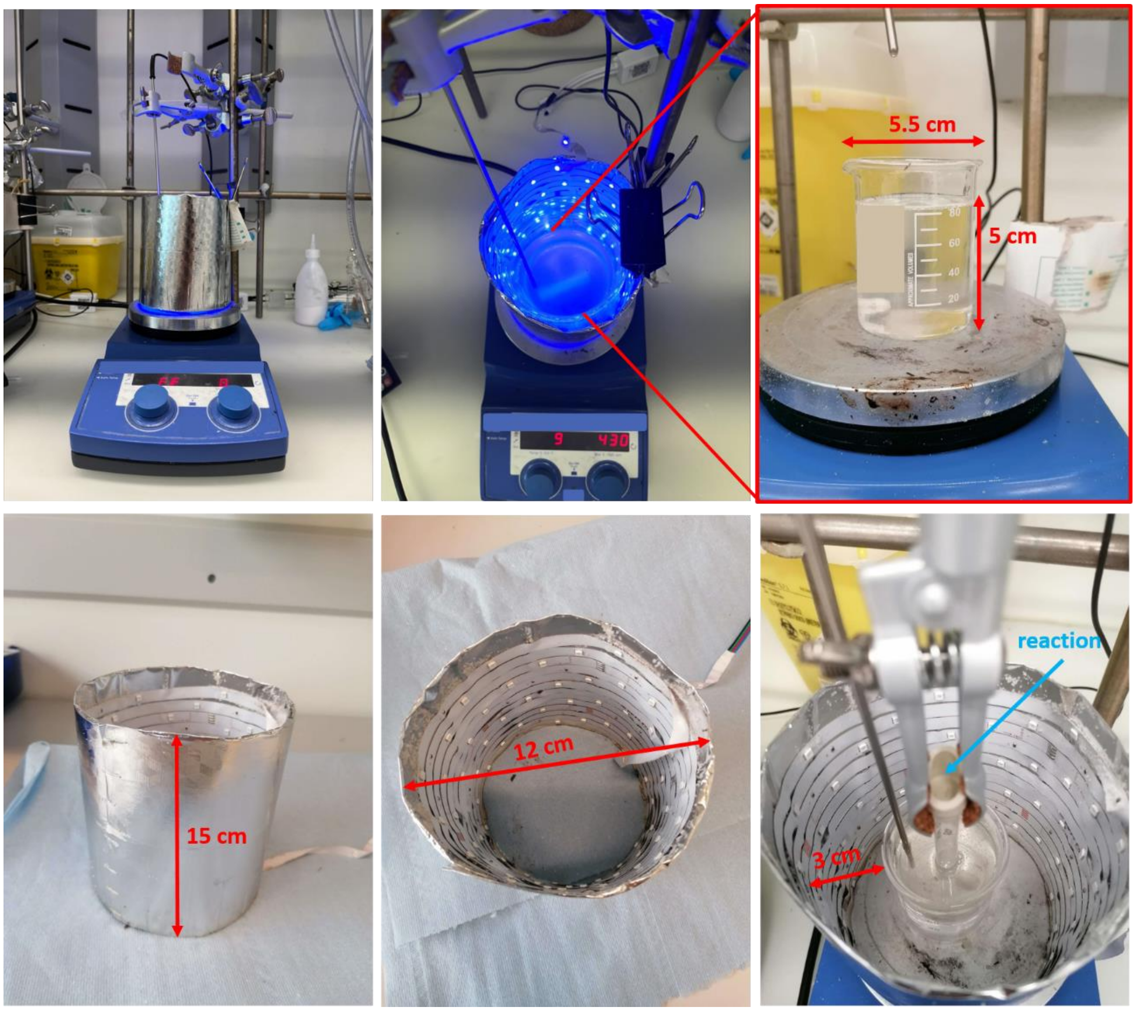

Figure S4: Digital image of the experimental set-up for the depolymerization.

General procedure for depolymerization

The quantities of reagents in this procedure refer to depolymerization using 1 equiv. of catalyst. In a 15 ml glass vial 2 mg, (1. 58 x 10^{-5} mol) of $FeCl_2$ and 5.09 mg (1. 58 x 10^{-5} mol) of tetra butyl ammonium bromide (TBABr) were introduced and the vial was sealed immediately with a rubber septum, to prevent oxidation of the catalyst and the vial was purged with nitrogen to remove any residual oxygen. In a separate vial, 15 ml of dichlorobenzene (DCB) and 150 µl of poly(ethylene glycol) methyl ether with average M.W. 350, as an internal standard, were deoxygenated. Upon deoxygenation of both vials, 12.3 ml of deoxygenated DCB was added to the vial that was containing the catalyst, under nitrogen. The vial was filled with nitrogen and sonicated for 3 minutes to make sure of catalyst solubilization, resulting in an almost colorless (slightly yellow color) catalyst solution. This stock solution was kept under nitrogen to prevent any potential oxygen diffusion and oxidation. In parallel, 9.1 mg (1.28 x 10^{-6} mol, 1 equiv.) of PBzMA was weighed in a 10 ml glass tube, equipped with a stirring bar, and sealed with a rubber septum and covered with parafilm. The vial was purged with nitrogen to remove the oxygen. Next, under nitrogen, 1 ml of catalyst solution (0.1625 mg $FeCl_2$, 1.28 x 10^{-6} mol, 1 equiv. and 0.4133 mg TBABr, 1.28 x 10^{-6} mol, 1 equiv.) was transferred in the tube congaing the polymer and the solution was stirred under nitrogen to ensure full polymer solubilization (9.1 mg/ml polymer concentration). Before immersing the reaction tube in the photoreactor (**Figure s4**), a time 0 sample was taken for ^{1}H NMR analysis. The reaction tube was placed in a pre-heated oil bath in the photoreactor with the light on. For different catalyst concentrations, the ratios altered, keeping the same procedure.

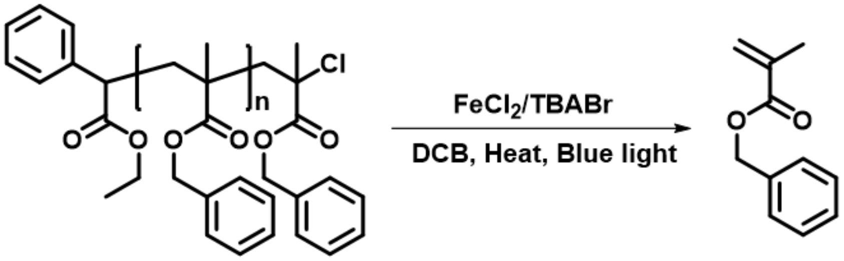

Scheme S2: Chemical illustration of depolymerization of poly(benzyl methacrylate) via photocatalytic Fe-ATRP depolymerization.

Depolymerization

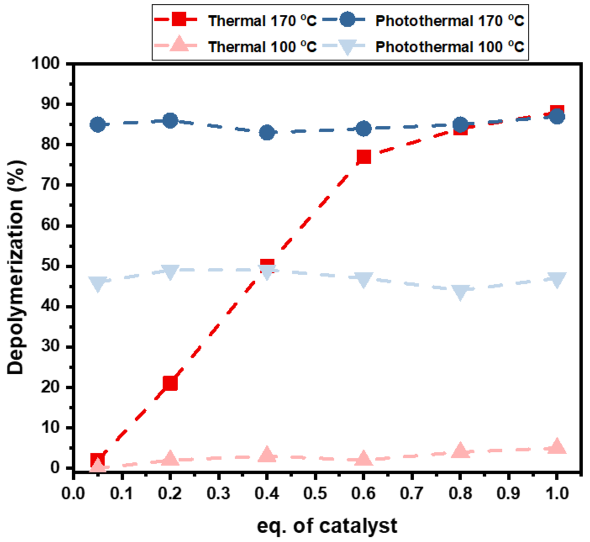

Figure S5: Depolymerization of PBzMA using different catalyst equivalents at 100 and 170 °C in thermal (red) and photothermal (blue) system. The equivalents of catalyst are calculated in regards to chain end group. For example 1 eq of catalyst means [Polymer]:[$FeCl_2$]:[TBABr] = [1]:[1]:[1]. The repeat unit concentration was 50 mM and the samples were taken after 1 h of reaction.

Table S3: Depolymerization of PBzMA using different catalyst equivalents at 100 and 170 °C in thermal (red) and photothermal (blue) systems. The equivalents of catalyst are calculated in regards to the chain end group. For example, 1 eq of catalyst means [Polymer]:[$FeCl_2$]:[TBABr] = [1]:[1]:[1]. The repeat unit concentration was 50 mM and the samples were taken after 1 h of reaction.

**Eq. of catalyst**	**Thermal (170 °C)**	**Photothermal (170 °C)**	**Thermal (100 °C)**	**Photothermal (100 °C)**
**4**	**83**	**85**	**5**	**47**
**1**	**88**	**87**	**4**	**44**
**0.8**	**84**	**85**	**2**	**47**
**0.6**	**77**	**84**	**2**	**49**
**0.4**	**50**	**83**	**3**	**49**
**0.2**	**21**	**86**	**2**	**46**
**0.05**	**2**	**85**	**0**	**48**
**0.0125**	**-**	**26**		
**0.00625**	**-**	**10**		

Table S4: SEC analysis of PBzMA after depolymerization reaction under various catalyst concentrations at 170 °C. [Polymer]:[$FeCl_2$]:[TBABr] = [1]:[x]:[x]. The repeat unit concentration was 50 mM, and the samples were taken after 1 h of reaction.

**Equiv. of catalyst**	M_n **(SEC)**	*Đ*
0	7200	1.18
1	6400	1.23
0.8	6500	1.22
0.2	6500	1.22
0.05	6500	1.22

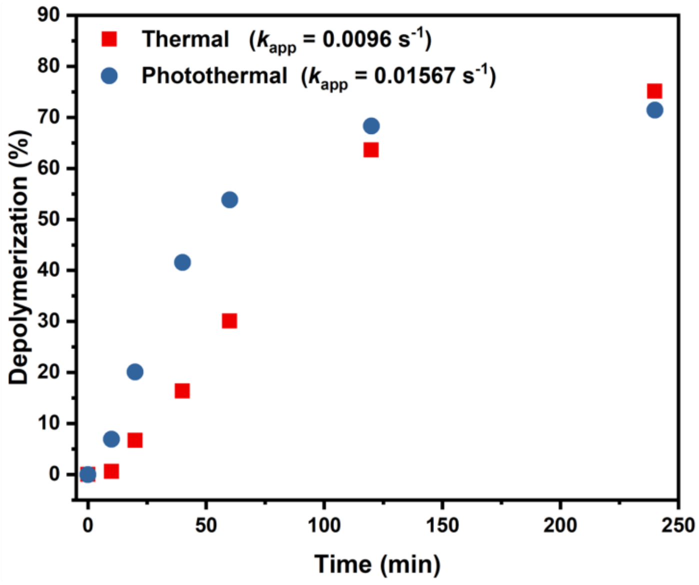

Figure S6: Merged thermal (red) and photothermal (blue) depolymerization kinetics of PBzMA. The repeat unit concentration was 50 mM and the reaction temperature 120 °C. [Polymer]:[$FeCl_2$]:[TBABr] = [1]:[0.05]:[0.05].

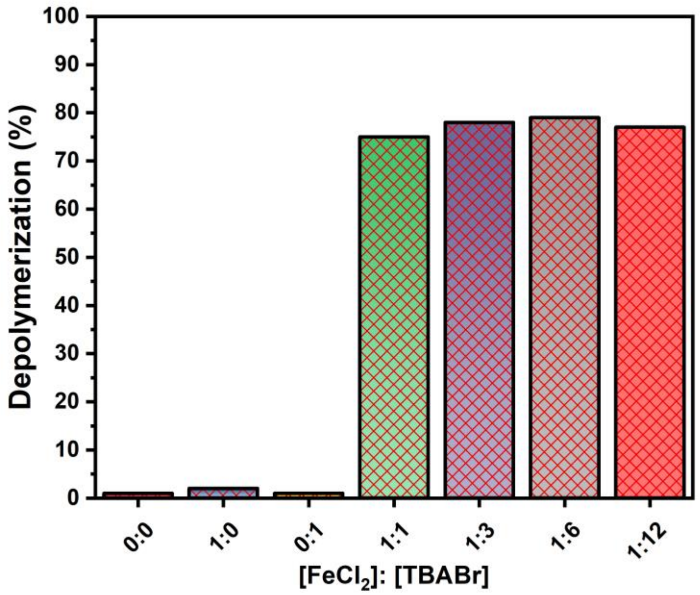

Figure S7: Control experiments in photocatalytic depolymerization of PBzMA. The repeat unit concentration was 50 mM and the samples were taken after 1 h of reaction at 170 °C and under blue light irradiation using 0.05 equivalent of $FeCl_2$.

Table S5: Control experiments in photocatalytic depolymerization of PBzMA. The repeat unit concentration was 50 mM and the samples were taken after 1 h of reaction at 170 °C and under blue light irradiation using 0.05 equivalent of $FeCl_2$.

$FeCl_2$: TBABr	Depolymerization (%)
**0:0**	**0**
**1:0**	**2**
**0:1**	**0**
**1:1**	**75**
**1:3**	**78**
**1:6**	**79**
**1:12**	**78**

Table S6: Depolymerization of PBzMA at different temperatures using 1 equiv. of catalyst. [Polymer]:[$FeCl_2$]:[TBABr] = [1]:[1]:[1] in DCB at 50 mM repeat unit concentration. The reactions at 170, 150 and 120 °C were stopped after 1 h , the reaction at 100 °C left for 2 h* after which the monomer yield did not increase, while the reaction at 80 °C was left for 24 h.

Temperature (°C)	Thermal Depolymerization (%)	Photothermal Depolymerization (%)
170	88	90
150	81	86
120	71	75
100*	6	54
80**	0	15

Table S7: Depolymerization of PBzMA at different temperatures using 0.05 equiv. of catalyst. [Polymer]:[$FeCl_2$]:[TBABr] = [1]:[0.05]:[0.05] in DCB at 50 mM repeat unit concentration. The reactions were stopped after 1 h exept the reaction at 100 °C, which was left for 2 h*.

Temperature (°C)	Thermal Depolymerization (%)	Photothermal Depolymerization (%)
170	5	84
150	0	82
120	0	80
100*	0	59

Table S8: Temporal control of photocatalytic depolymerization of PBzMA under the following condition: [Polymer]:[$FeCl_2$]:[TBABr] = [1]:[1]:[1] in DCB at 50 mM repeat unit concentration at 100 °C.

Light (on/off)	Time (min)	Depolymerization (%)
ON	0-20	18
OFF	20-40	31
ON	40-70	44
OFF	70-100	45
ON	100-160	44
OFF	160-210	45

Table S9: Temporal control of photocatalytic depolymerization of PBzMA under the following condition: [Polymer]:[$FeCl_2$]:[TBABr] = [1]:[0.05]:[0.05] in DCB at 50 mM repeat unit concentration at 100 °C.

Light (on/off)	Time (min)	Depolymerization (%)
ON	0-20	1
OFF	20-40	3
ON	40-70	12
OFF	70-100	12
ON	100-160	24
OFF	160-210	24

Table S10: Photocatalytic depolymerization kinetic experiments of PBzMA at 120 °C, utilizing 0.05 eq of $FeCl_2$ and $FeCl_3$. [Polymer]:[$FeCl_2$ or $FeCl_3$]:[TBABr] = [1]:[0.05]:[0.05] in DCB at 50 mM repeat unit concentration.

Time (min)	Depolymerization (%) using $FeCl_2$	Depolymerization (%) using $FeCl_3$
0	0	0
10	7	0
20	20	5
40	42	19
60	54	31
120	68	58
240	71	71

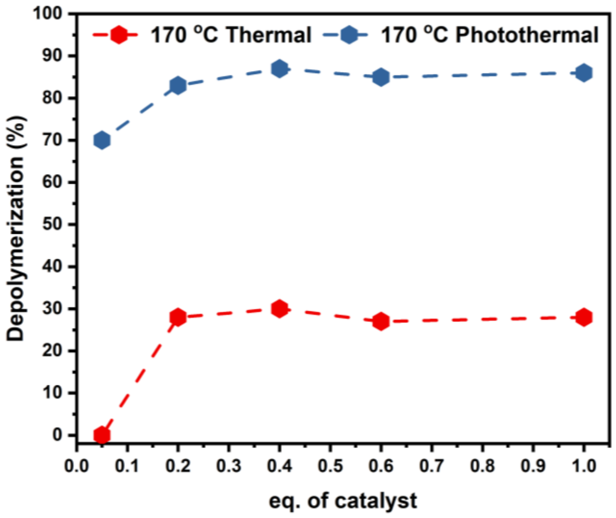

Figure S8: Depolymerization of PBzMA using different catalyst equivalents at 170 °C in thermal (red) and photothermal (blue) system. The equivalents of catalyst are calculated in regards to chain end group. For example 1 eq of catalyst means [Polymer]:[$FeCl_3$]:[TBABr] = [1]:[1]:[1]. The repeat unit concentration was 50 mM and the samples were taken after 1 h of reaction.

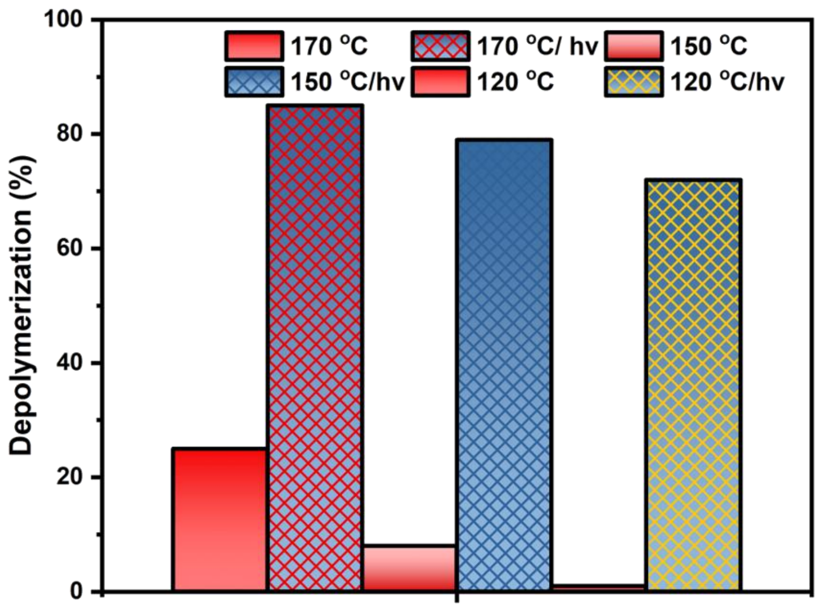

Figure S9: Depolymerization of PBzMA at different temperatures using 1 equiv. of catalyst. [Polymer]:[$FeCl_3$]:[TBABr] = [1]:[1]:[1] in DCB at 50 mM repeat unit concentration. The reactions were stopped after 1 h.

Table S11: Depolymerization of PBzMA at different temperatures using 1 equiv. of catalyst. [Polymer]:[$FeCl_3$]:[TBABr] = [1]:[1]:[1] in DCB at 50 mM repeat unit concentration. The reactions were stopped after 1 h.

**Temperature (°C)**	**Thermal Depolymerization (%)**	**Photothermal Depolymerization (%)**
**170**	**25**	**85**
**150**	**8**	**79**
**120**	**1**	**72**

Table S12: Depolymerization of PBzMA at different temperatures using 0.05 equiv. of catalyst. [Polymer]:[$FeCl_3$]:[TBABr] = [1]:[0.05]:[0.05] in DCB at 50 mM repeat unit concentration. The reactions were stopped after 1 h.

**Temperature (°C)**	**Thermal Depolymerization (%)**	**Photothermal Depolymerization (%)**
**170**	**5**	**70**
**150**	**1**	**65**
**120**	**1**	**63**

Table S13: Temporal control of photocatalytic depolymerization of PBzMA under the following condition: [Polymer]:[$FeCl_3$]:[TBABr] = [1]:[0.05]:[0.05] in DCB at 50 mM repeat unit concentration at **170 °C**.

Light (on/off)	Time (min)	Depolymerization (%)
ON	0-3	5
OFF	3-23	6
ON	23-25	17
OFF	25-55	19
ON	55-65	23
OFF	65-125	24

Table S14: Temporal control of photocatalytic depolymerization of PBzMA under the following condition: [Polymer]:[$FeCl_3$]:[TBABr] = [1]:[0.05]:[0.05] in DCB at 50 mM repeat unit concentration at **150 °C**.

Light (on/off)	Time (min)	Depolymerization (%)
ON	0-10	25
OFF	10-20	27
ON	20-25	39
OFF	25-63	46
ON	63-69	55
OFF	69-129	59
ON	129-189	62

Table S15: Temporal control of photocatalytic depolymerization of PBzMA under the following condition: [Polymer]:[$FeCl_3$]:[TBABr] = [1]:[0.05]:[0.05] in DCB at 50 mM repeat unit concentration at **120 °C**.

Light (on/off)	Time (min)	Depolymerization (%)
ON	0-20	19
OFF	20-40	19
ON	40-70	45
OFF	70-100	45
ON	100-160	69
OFF	160-220	70

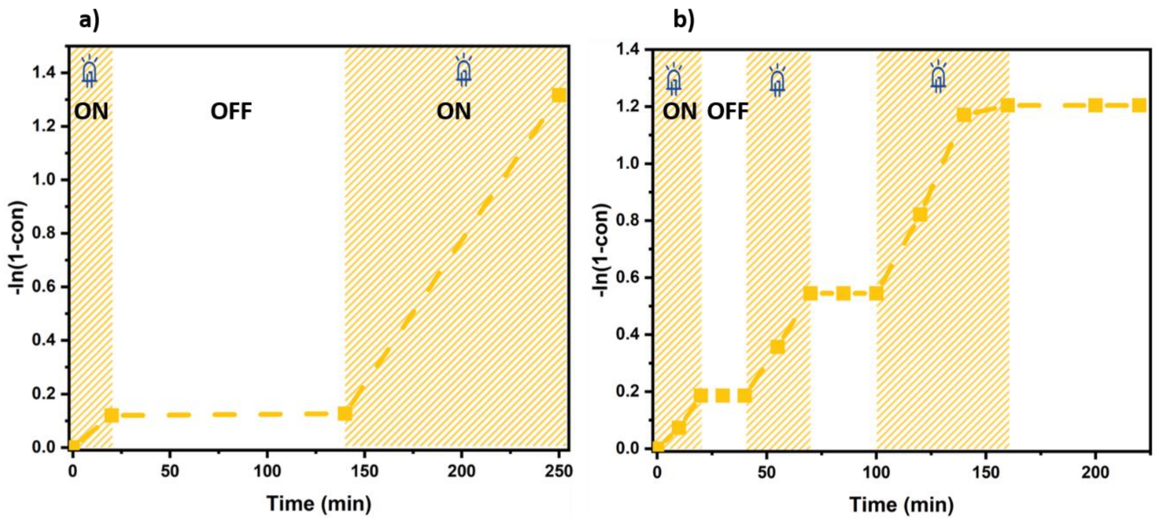

Figure S10: Temporal control experiments at 120 °C, utilizing the following conditions : [Polymer]:[$FeCl_3$]:[TBABr] = [1]:[0.05]:[0.05] in DCB at 50 mM repeat unit concentration. Figure a) demonstrates a long first "off" period of 120 min, without any detectable monomer generation and b) demonstrates a temporal control experiment with multiple samples obtained during the reaction.

Table S16: In this experiment, samples were placed in the photoreactor without initially turning the light on. After the incubation of the reaction tubes at the given temperatures (170, 150, 120) for different periods of time (0, 20, 40 and 60 min) light was switched on, and the reaction was allowed to proceed for 1 more hour for all the experiments. Following condition were used: [Polymer]:[**$FeCl_3$**]:[TBABr] = [1]:[0.05]:[0.05] in DCB at 50 mM repeat unit concentration.

Thermal incubation time (min)	170 °C	150 °C	120 °C
0	83	78	74
20	30	62	74
40	26	52	74
60	24	45	72

Table S17: In this experiment, samples were placed in the photoreactor without initially turning the light on. After the incubation of the reaction tubes at the given temperatures (170, 150, 120) for different periods of time (0, 20, 40 and 60 min) light was switched on, and the reaction was allowed to proceed for 1 more h for all the experiments. Following condition were used: [Polymer]:[**$FeCl_2$**]:[TBABr] = [1]:[0.05]:[0.05] in DCB at 50 mM repeat unit concentration.

Thermal incubation time (min)	170 °C	150 °C	120 °C
0	80	74	76
20	41	66	73
40	26	51	74
60	17	34	73

Table S18: Photocatalytic depolymerization of PnBMA and $PMMA_{51}$ for 1 h. Following condition were used: [Polymer]:[$FeCl_2$]:[TBABr] = [1]:[0.05]:[0.05] in DCB at 50 mM repeat unit concentration.

Polymer	Temperature (°C)	Depolymerization (%)
$PnBMA_{38}$	120	71
$PMMA_{51}$	120	70
$PMMA_{51}$	170	86

Table S19: Photocatalytic depolymerization of PBzMA with green light. Following condition were used: [Polymer]:[$FeCl_2$]:[TBABr] = [1]:[0.05]:[0.05] in DCB at 50 mM repeat unit concentration. The reaction took place for 1h at 120 °C.

Light	Depolymerization (%)
**Green (λ_{max} = 510 ± 31 nm)**	**70**

Table S20: Photocatalytic depolymerization of PBzMA at different repeat unit concertation using 1 eq. of $FeCl_2$. The samples were taken after 1 h of reaction at 170 °C.

Repeat unit concentration (mM)	Depolymerization (%)
**50**	**83**
**250**	**71**
**500**	**68**
**750**	**58**
**1000**	**56**
**1500**	**54**
**2000**	**48**
